# The Role of M6A LncRNA Modification in Papillary Thyroid Cancer

**DOI:** 10.3390/ijms26072833

**Published:** 2025-03-21

**Authors:** Michelle S. Klausner, Caylee A. Greenberg, Kaleb A. Noruzi, Raj K. Tiwari, Jan Geliebter

**Affiliations:** 1 School of Medicine, New York Medical College, Valhalla, NY 10595, USA; mklausne2@student.nymc.edu (M.S.K.);; 2 Department of Pathology, Microbiology, and Immunology, and Department of Otolaryngology, New York Medical College, Valhalla, NY 10595, USA

**Keywords:** thyroid cancer, papillary thyroid cancer, LncRNA, N6-methyladenosine, epigenetics, m6A modification, cancer pathogenesis

## Abstract

Thyroid Cancer (TC) is the most common endocrine cancer, of which papillary thyroid cancer (PTC), a well-differentiated type of TC, accounts for 80–90%. Long non-coding RNAs (lncRNAs), which comprise non-protein-coding segments of the genome, have been found to play a crucial role in various biological processes, including cancer development. The activity of lncRNAs is modified through epigenetic modifications, with N6-Methyladenosine (m6A) modifications implicated in the progression of several malignancies. The activity of m6A is further regulated by modifying enzymes classified as “readers”, writers”, and “erasers”, of which specific enzymes have been found to play a role in various aspects of PTC. Recent research has highlighted the significance of m6A modification in regulating the expression and function of lncRNAs associated with PTC pathogenesis. Dysregulation of this process implicates tumor proliferation, invasion, and metastasis, with subsequent impact on prognosis. Therefore, understanding the interplay between m6A modification and lncRNAs provides valuable insights into the molecular mechanisms underlying PTC progression. This narrative review aims to explore the established role of several prominent m6A modifying enzymes and lncRNAs on cancer pathogenesis and seeks to clarify the function of these enzymes in PTC pathogenesis.

## 1. Introduction

Thyroid cancer (TC) is the most common endocrine cancer and accounts for 1–2% of malignancies worldwide [1]. Papillary thyroid cancer (PTC) is estimated to account for 80–90% of TC, with approximately 10% of PTC metastatic at presentation [1,2,3]. While the 5-year survival of non-metastatic PTC is over 99% after surgery, that number decreases to 51.9% with the presence of distant metastases [3,4,5]. Additionally, the recurrence rate of PTC has been found to be as high as 35%, with the number including patients with low-risk tumors that recur due to the multifocality and early lymph node metastasis of PTC [6]. Given that PTC typically occurs in younger females, and recurrences occur any time between years and decades following the primary cancer identification, the identification of tumors that may be likely to recur remains of clinical importance. This underscores the necessity of identifying individuals with higher-risk PTC phenotypes to implement more aggressive treatment measures, while simultaneously identifying lower-risk patients to prevent overtreatment. 

The most aggressive form of thyroid malignancy is anaplastic thyroid cancer (ATC), which despite only comprising 2–5% of all TC, accounts for 14–50% of TC deaths, with a median survival of 5 months [7]. While findings have been at times inconclusive, there is growing evidence to suggest that ATC arises from PTC [8]. A DNA sequencing study by Quiros et al. exploring genetic mutations in eight ATC samples concluded that many ATCs that have papillary components are derived from mutated PTC [9]. Additional studies exploring the differentiation status of ATC compared with PTC have unsurprisingly found that ATC cells are moderately differentiated, compared with most PTC cells, which remained in a differentiated state [10]. Given the potential for some PTCs to progress to ATC, though most do not, identifying factors that may signify a potential to progress is critical. Similarly, it is essential to expand upon current models of cancer classification as knowledge of the genomic compositions of cancers and pathways important to cancer prognostication advances.

Several classification systems have been established to characterize tumor behavior and to identify individuals with higher-risk variants of PTC. Generally, the determination of tumor stage has largely relied on the TNM classification system, which stages PTC into stages I–IV depending on patient age, tumor size, nodal metastases, and distant metastases [11]. As molecular tumor testing has advanced, further attempts to characterize tumors and identify markers of aggressive behavior have emerged. One such method includes the utilization of a thyroid differentiation score (TDS), created by The Cancer Genome Atlas to assign a molecular classification system to PTC and identify molecular markers indicative of advanced clinical disease, and thus individuals at higher risk of recurrence [12]. The TDS is a list of 16 genes involved in thyroid metabolism and function that are used to evaluate the differentiation status of TC. Several other studies are now examining the roles of pathways such as *BRAF* and *Ras* in TC progression and severity, which may pave the way for further models of cancer classification and prognostication in the future.

While the TNM tumor staging system and TDS are used to guide tumor classification, the role of long non-coding RNA (lncRNA) in impacting the clinicopathologic characteristics of malignancies including PTC is being explored. LncRNAs are defined as non-protein coding regions of the transcriptome that are over 200 nucleotides in length [13]. Several roles for lncRNA have been described, including in genome organization, cell structure, gene expression, and protein interaction [13]. The role of lncRNA in the modulation of cancer, including PTC, has emerged as an important player in tumor behavior. Several lncRNAs have been identified in relation to PTC, displaying both tumor-suppressive and oncogenic roles that are implicated in thyroid carcinogenesis [14,15,16,17].

One way of modulating the function of lncRNAs is through epigenetic modification. Epigenetic modifications refer to traits that are external to the genetic sequence, and are involved in altering gene expression through changes in DNA availability and chromatin pattern [18]. Epigenetic modifications are often influenced by the environment, though they may be heritable, and provide for ‘communication’ between the genome and external factors [18]. Several key epigenetic modifications exist, which include the following: DNA methylation, histone modification, and nucleosome positioning [19]. DNA methylation involves covalently attaching a methyl group to cytosine on the C5 position, to form 5-methylcytosine [20]. DNA methylation occurs mainly in CpG dinucleotide-containing areas of the genome and frequently occurs in regulatory regions that affect gene expression [18]. In turn, DNA methylation results in transcriptional repression by preventing the binding of transcription factors or by promoting the binding of transcriptional repressors [18]. In cancer, DNA methylation plays a role by implicating the expression of tumor suppressor genes, oncogenes, and other regulatory genes, thereby influencing the environment to allow the development, or progression, of malignancy [21]. Histone modification may involve methylation, acetylation, phosphorylation, and ubiquitination, which all lead to a change in the structure of chromatin and thus the expression of certain genes [18]. Histone post-translational modification has similarly been implicated in disease processes including cancer, with several characterized modifications deregulated in certain malignancies [22]. Lastly, nucleosome positioning helps determine chromatin structure, and depending on the spacing of nucleosomes it may render areas of the genome as transcriptionally active or repressed [18]. Similarly, the role of nucleosome spacing has been found to be of importance for several malignancies, including breast and colorectal cancer [23,24]. One of the most important modifications identified to play a role in PTC is DNA methylation. Furthermore, lncRNAs can be epigenetically modified through enzymes that are broadly divided into three classes: “writers”, “readers”, and “erasers” [25]. The role of these enzymes has been well established in many cancers such as esophageal squamous cell carcinoma, osteosarcoma, breast cancer, and more. One of the most commonly modified nucleosides found in lncRNA is N6-methyladenosine (m6A), which adds a methylation of the sixth nitrogenous base of adenylated RNA or DNA to affect their stability, splicing, and translation [25,26]. M6A has been extensively characterized in several cancers, and research has highlighted the significance of m6A modification in regulating the expression and function of lncRNAs associated with PTC pathogenesis.

This narrative review will provide an overview of the current literature on the role of m6A methylation and lncRNAs in tumorigenesis. We aim to examine various m6A methylation regulators: writers, readers, and erasers, which are implicated in PTC progression, providing a comprehensive overview of the major studies and their findings to date. By analyzing these enzymes’ impact on PTC, we aim to offer valuable prognostic insights. Furthermore, this review explores the specific mechanisms by which m6A modifications influence PTC development and progression, highlighting the interplay between epigenetic alterations and cancer pathogenesis. Through the review, we aim to clarify the complex molecular landscape of PTC and identify potential avenues for diagnostic and therapeutic interventions.

## 2. Role of M6A Methylation and LncRNA in Cancer Pathogenesis

N-6 methyladenosine (m6A) is the most common methylated nucleoside in eukaryotic mRNA [27,28]. M6A is produced through methylation of the sixth nitrogenous base of adenylated DNA or RNA [29] by methyltransferases, known as “writers” [30]. This modification is present in many forms of RNA, including mRNA, rRNA, lncRNA, miRNA, circRNA, and snRNA [31,32]. M6A modification and function are regulated by the interplay of methyltransferases (“writers”), demethylases (“erasers”), and proteins known as “readers” that recognize and bind to m6A, subsequently triggering various mechanisms of RNA processing and metabolism [31,33]. The writers mainly consist of METTL3, METTL14, WTAP, KIAA1429, METTL16, RBM15, and ZC3H13. FTO and ALKBH5 compose the erasers, while YTHDF1/2/3, YTHDC1/2, HNRNPA2B1, HNRNPC11, and HNRNPG compose the readers [33,34]. Innate regulation of m6A by writers, erasers, and readers is crucial to proper cellular function, including regulation of transcription, alternative splicing, mRNA translation, export, and degradation, genomic stability, apoptosis, autophagy, cell migration and stress adaptation, cell–cell adhesion, and numerous intracellular signaling pathways [32,33,35,36]. Through its fundamental role in cellular functionality, m6A exerts effects on many physiological processes, including stem cell pluripotency and differentiation, immune response [35], neuronal maturation and synaptic transmission, learning and memory [32], spermatogenesis [37], embryogenesis [38], among others [32]. Conversely, perturbations of the m6A regulatory axis are implicated in pathological processes such as cancer, tumorigenic, and occasionally tumor suppressive roles. These perturbations are primarily due to upregulation or downregulation of the writer, eraser, and reader proteins involved in m6A regulation, and are found in a plethora of cancers [31,33,36,39]. Aberrant expression of m6A-related proteins has been shown to promote activation of oncogenic signaling pathways, including but not limited to the MYC, Wnt/B-Catenin, PI3K/AKT/mTOR, and p53 pathways, leading to tumor growth, stemness, invasion, metastasis, drug resistance, and immune evasion [31]. An example of a mechanism through which aberrant expression of these proteins promotes oncogenesis is overexpression of METTL3, a methyltransferase or writer, in acute myeloid leukemia (AML). Increased expression of METTL3 and subsequent increased m6A methylation of the target genes, *MYC*, *BCL2*, and *PTEN*, leads to increased translation of these genes, resulting in retention of cell pluripotency and inhibition of differentiation and apoptosis [39].

The majority of the human genome is transcribed into non-coding RNA (ncRNA) [40,41,42,43], and the most prevalent form of ncRNA is lncRNA [13,43]. lncRNA has been determined to have extensive involvement in the regulation of chromatin modification, transcription, and translation, along with other various cellular functions, through diverse interactions with DNA, RNA, and protein [13,43]. For example, lncRNA naturally recruits and forms complexes with transcription factors or histone or chromatin-remodeling proteins and then interacts with target histones or genomic loci, such as promoters, to epigenetically modulate gene expression [40,42]. The widespread impact of lncRNA on genome organization and gene expression allows for considerable vulnerability to genomic deregulation and the development of disease processes, like cancer, when there is dysfunction or abnormal expression or epigenetic modulation of lncRNA [41,42]. Abnormally elevated expression of lncRNA can lead to increased transcription and expression of genes promoting cell survival, proliferation, invasion, and other oncogenic qualities [40,42]. For example, the lncRNA *HOTAIR*, *PSMG3-AS1*, and *BHLHE40-AS1* have been shown to be overexpressed in breast cancer, leading to tumorigenesis and disease progression through various mechanisms [44,45,46]. *HOTAIR* has been shown to increase the activity of the AKT signaling pathway and promote proliferation, invasion, and migration [44]. *PSMG3-AS1* has been shown to facilitate cancer cell amplification and migration through regulation of the expression of COL1A1 [45]. *BHLHE40-AS1* is reported to mediate IL-6 and STAT3 signaling and the resultant tumorigenic inflammatory microenvironment [46].

In addition to elevated expression of lncRNA, epigenetic modulation of lncRNA has been found to be associated with various cancers [25,47]. Epigenetic modification of DNA encoding lncRNA has been demonstrated to play an important part in tumorigenesis [25,47]. Wang et al. showed that epigenetic activation and silencing of lncRNAs through DNA hypomethylation and hypermethylation, respectively, underlies their role in tumorigenesis. Certain lncRNAs that are highly expressed in cancers, such as *EPIC1*, *SNHG12*, and *MINCR*, were determined to be epigenetically activated by DNA hypomethylation. On the other hand, certain lncRNAs that are underexpressed in cancers, such as *SNHG18* and *AF186192.1*, were revealed to be epigenetically silenced via DNA hypermethylation [47].

Direct epigenetic modification of lncRNA via methylation is also implicated in numerous instances of tumorigenesis [25]. The main forms of lncRNA epigenetic modifications that are associated with cancer are the production of 5-methylcytosine (m5c) and m6A through methylation by methyltransferases or “writers”, as previously mentioned [25,31,33]. NSUN2 is the writer responsible for the production of m5c in lncRNA and is implicated in tumorigenesis through variegated mechanisms [25,48]. In esophageal squamous cell carcinoma (ESSC), overexpression of the lncRNA, *NMR*, is associated with increased metastasis and poor survival. *NMR* overexpression results in increased m5c methylation of *NMR* by SUN2, which may competitively inhibit m5c methylation of various other mRNAs by SUN2. Methylation of mRNA by NSUN2 generally increases its stability and translation, so decreased m5c methylation of mRNA in ESSC may decrease the translation of various proteins and ultimately contribute to increased tumor metastasis and poor survival [49]. In hepatocellular carcinoma (HCC), upregulation and increased m5c methylation by NSUN2 are associated with poor differentiation of cancer cells. Sun et al. showed that H19 with m5c methylation binds to the oncoprotein, G3BP1, leading to the accumulation of MYC and tumorigenesis [50]. ALYREF and YBX1 are m5c readers that have been determined to have involvement in tumorigenesis [25]. ALYREF is overexpressed in breast cancer and was shown by Klec et al. to bind and regulate *NEAT1* lncRNA, which is known to be overexpressed in breast cancer and associated with metastasis and poor survival [51]. Elevated expression of *DSCAM-AS1* in breast, prostate, and lung cancer is correlated with poor prognosis, and Zhang et al. demonstrated that YBX1 reads and interacts with *DSCAM-AS1* and subsequently regulates expression of the FOXA1 oncogene [52].

M6A modification of lncRNA has also been discovered to be involved in the development of cancer and is arguably more extensively involved than m5c modification. m6A-modified lncRNA (mML) acts to drive oncogenesis through many different mechanisms centered around the action of the writer, eraser, and reader proteins [25]. One notable mechanism involving writers consists of m6A-methylation of lncRNA that is overexpressed in cancer, such as *NEAT1*, which is overexpressed in many cancers [48]. Wen et al. showed that increased m6A-methylation of *NEAT1*, which is likely a natural consequence of *NEAT1* overexpression, increases prostate cancer metastasis and decreases survival through m6A-dependent induction and recruitment of CYCLINL1 and CDK19 to the RUNX2 promoter, leading to increased RUNX2 expression [53]. Another important mechanism revolves around the effects of m6A-methylation of lncRNA by METTL3. METTL3 methylation of several lncRNAs, namely *LINC00958*, *FAM225A*, and *RP11*, increases their stability and results in upregulation and subsequent tumorigenesis in HCC, nasopharyngeal carcinoma, and colorectal cancer, respectively [54]. Demethylation of mML by the erasers, ALKBH5 and FTO, can result in both tumorigenesis and tumor suppression in different cancers [25,54]. ALKBH5 is downregulated in pancreatic cancer, in which it normally functions to decrease m6A-methylation of lncRNA *KCNK15-AS1*, leading to decreased tumor motility [55]. Conversely, ALKBH5 demethylation suppresses the degradation of lncRNA *PVT1*, which is upregulated in osteosarcoma and promotes proliferation [56]. FTO has been reported to be downregulated in glioblastoma (GBM) and gastric cancer [57] and its presence is associated with a favorable prognosis in GBM [58]. FTO has also been reported to mediate resistance of pancreatic ductal adenocarcinoma to gemcitabine through stabilization of lncRNA *LINC01134*, which is upregulated in this cancer and is associated with gemcitabine resistance [59]. The m6A reader, IGF2BP2, has been shown to be upregulated in pancreatic cancer and increases the expression of mML *DANCR*, which promotes proliferation and cancer stemness [60]. Another reader, YTHDF3, promotes tumorigenesis in colorectal cancer through the degradation of mML *GAS5*, which normally negatively regulates the YAP oncogene [61]. Other readers, HNRNPC and YTHDF2, are upregulated and associated with poor prognosis in several cancers [58,62]. 

The accumulating body of evidence indicating the cruciality of mML in cancer progression has led to widespread awareness of the need to develop diagnostic, prognostic, and therapeutic modalities based on the presence or absence of specific mMLs in cancers. Many research efforts have created prognostic risk assessment models based on the signature of mMLs present in specific cancers [25,54]. Cusenza et al. summarized these models [25]. They reported the discovery of mML signatures associated with hematologic malignancy, namely AML, and the solid tumors of osteosarcoma, glioma, pancreatic ductal carcinoma, clear-cell renal carcinoma, lung adenocarcinoma, colon adenocarcinoma, HCC, and breast, gastric, ovarian, and bladder cancer [25]. There have also been several research efforts focused on the development of novel treatments that target oncogenic m6A regulators, namely ALKBH5 and FTO [63]. It is important to note that despite the wealth of research identifying and characterizing mML signatures in numerous cancers, there is minimal research on mMLs in specific TCs, particularly PTCs.

## 3. Involvement of M6A Methylation Regulators in Papillary Thyroid Cancer: Prognostic Values and Relationship to LncRNA

While our understanding of the significance of m6A-associated lncRNAs in cancer pathogenesis has become more established, research into the role of m6A RNA methylation regulators and m6A-associated lncRNAs in PTC specifically remains limited. Studies over the last few years have begun to elucidate m6A methylation regulators that are differently expressed in PTC. Further, several of these m6A methylation regulators have been shown to be related to prognosis and overall survival. Several studies have created their own risk signatures using these m6A RNA methylation regulators to estimate prognosis, and some have evaluated the relationship between m6A RNA regulators and lncRNA that are thought to be involved in PTC. A summary of these findings can be found in Table 1.

### 3.1. M6A Modifying Readers

#### 3.1.1. HNRNPC

The m6A “reader” HNRNPC has been studied in various malignancies such as non-small cell lung cancer, prostate cancer, breast cancer, gastric cancer, and more [64,65,66]. Studies in these malignancies have found a general correlation demonstrating that higher expression levels of HNRNPC are associated with a poorer prognosis and poorer therapeutic response [64,66]. Several studies have found HNRNPC to be upregulated in PTC [67,68,69,70]. However, the relationship between HNRNPC and PTC prognosis has not been consistent. Hou et al. found that HNRNPC was overexpressed in PTC tissue in comparison to normal control samples, but it was not significantly associated with overall survival [67]. X Wang et al. also noted HNRNPC to be upregulated in PTC, though not significantly, and did not find a significant association with disease-free survival [68]. While these two studies did not find a significant relationship between HNRNPC and prognosis, N Xu et al. did find HNRNPC to be significantly related to poor prognosis of differentiated thyroid cancer (DTC) and included HNRNPC in their risk signature [69]. Notably, DTC includes both PTC and follicular thyroid cancer which may involve different prognostic factors. In a study by W Wang et al., HNRNPC was found to be upregulated but was only positively correlated with the expression of 3 out of 19 m6A-related lncRNAs analyzed [70]. The authors commented on the overall prognostic value of m6A regulators but did not comment on HNRNPC specifically. Taken together, studies have been consistent in demonstrating the upregulation of HNRNPC in PTC. However, despite the established and significant role HNRNPC plays in other malignancies, studies have been unconvincing in describing a role or a significant relationship between the reader and PTC prognosis, with findings at times contradictory. This necessitates further studies to clarify the implication of HNRNPC in PTC prognosis and progression, if any.

#### 3.1.2. IGF2BP2

IGF2BP2 is also an RNA-binding protein or “reader” that has been studied in cancers such as glioma, ovarian, prostate, pancreatic, breast, and many more [71]. In these malignancies, IGF2BP2 levels have been associated with tumor progression, poorer prognosis, and lower overall survival [72,73]. The role of IGF2BP2 has also been described in PTC, and studies have found that it is generally upregulated in PTC and is involved in PTC differentiation and metastasis [68,70,74,75]. One study found that IGF2BP2 may work to promote tumor growth in PTC via interactions with *APOE* mRNA and the IL-6/JAK2/STAT3 pathway [74]. Sa et al. showed that IGF2BP2 stabilizes the transcription factor RUNX2, blocking the differentiation of PTC [75]. A study by W Wang et al. found that IGF2BP2 plays a role in lymph node metastasis via stabilization of DPP4 and activation of the NF-kB pathway [76]. Additionally, these researchers found that IGF2BP2 knockdown increased sensitivity to cisplatin whereas overexpression of IGF2BP2 decreased sensitivity to cisplatin. Another study found that IGF2BP2 may also be related to tyrosine kinase inhibitor resistance among PTC through modification of ERBB2 signaling and may be a target for improving resistance to these agents [77]. IGF2BP2 has also been found to be related to PTC prognosis. One study found IGF2BP2 to be the only m6A regulator related to disease-free survival out of all the m6A regulators studied [68]. These authors created their risk signature with IGF2BP2 as well as the genes *STT3A*, *MTHFD1*, and *GSTM4*, which were all strongly correlated with IGF2BP2 and disease-free survival. In terms of the relationship between IGF2BP2 and lncRNA in PTC, W Wang et al. found a positive correlation between the expression levels of IGF2BP2 in 9 out of 14 m6A-related lncRNAs analyzed [70]. Though the studies are limited, IGF2BP2 is likely involved in several modalities of PTC tumorigenesis, with its modulation potentially benefitting therapeutic success, and its expression assisting in prognostication.

#### 3.1.3. YTHDC2

YTHDC2 is a relatively newly identified member of the YT521-B homology (YTH) family [78]. The correlation between the expression levels of YTHDC2 and prognosis has been mixed: Lower expression levels were associated with poorer prognosis in lung and endocrine malignancies [79,80], higher expression levels were associated with a poorer prognosis and higher tumor stage in prostate and breast cancer [79,81], and confounding results were seen in gastric cancer [79,82]. The RNA binding protein YTHDC2 is downregulated in PTC [67,68,69,70,83]. Zhou et al. demonstrated that YTHDC2 inhibits the proliferation and apoptosis of PTC cells via cylindromatosis and inactivation of the Akt pathway [83]. Although the prognostic value of YTHDC2 has been evaluated in several studies, it has not been found to be related to PTC prognosis [67,68,69]. However, given that YTHDC2 is a newly discovered m6A binding protein, further studies may be needed to definitively elucidate its role in PTC prognosis and tumor stage.

### 3.2. M6A Modifying Writers

#### 3.2.1. METTL3

Research has shown that METTL3, a methyltransferase or “writer”, acts primarily as an oncogene in several cancers including breast cancer, leukemia, glioblastoma, and more, but has also been shown to have a role as a tumor suppressor in cancers such as renal cell carcinoma and bladder cancer [84]. METTL3 has been found to be decreased in PTC. METTL3 has been proposed to act as an inhibitor of PTC by stabilizing six transmembrane epithelial antigens of the prostate 2 (STEAP2) and suppressing the Hedgehog signaling pathway [85]. A separate study proposed that METTL3 acts to stabilize *ACSM5* mRNA [86]. *ACSM5* encodes an enzyme involved in fatty acid metabolism that has previously been implicated in the prognosis of lung adenocarcinoma [87]. In PTC, both METTL3 and *ACSM5* were found to have decreased expression levels, with lower levels associated with poorer prognosis [86]. He et al. demonstrated that METTL3 gene expression and protein levels were decreased in PTC tissue and that less METTL3 expression in PTC tissue was correlated with poorer prognosis [88]. This study also found that c-Rel and RelA act as downstream targets of METTL3, which inactivate the NF-κB pathway. When METTL3 activity was disrupted, there were increases in IL-8 secretion and recruitment of tumor-associated neutrophils. With IL-8 antagonists, tumor growth was slowed. A study by Ning et al. demonstrated how METTL3 may contribute to the tumor microenvironment [89]. They found that low METTL3 expression was correlated with a poor response to immune checkpoint blockade, whereas overexpression was associated with an improved response to anti-PD-1 therapy. Taken together, this research suggests that METTL3, or the downstream targets of METTL3 such as STEAP2, ACSM5, c-Rel, and RelA, may be potential therapeutic targets for PTC. However, while METTL3 consistently shows decreased expression levels in PTC across studies, several studies have also found that METTL3 was not related to PTC prognosis [67,68,69]. 

METTL3 was found to be related to 13 out of 19 lncRNAs researched by W Wang et al., one of which is the lncRNA *LINC00894*, a factor known to be involved in cancer [70]. Research into the relationship between METTL3 and lncRNA has been primarily with regard to *LINC00894* [90,91]. In one study, *LINC00894* was found to be a downstream target of METTL3, with increased METTL3 resulting in upregulation of *LINC00894* [91]. Upregulation of METTL3 or *LINC00894* caused decreased cell invasion, migration, viability, and colony formation, whereas inhibition of *LINC00894* caused increased cell proliferation and metastasis. Both METTL3 and *LINC00894* have been shown to be decreased in PTC [90,91]. Another study showed that *LINC00894* inhibits the lymphangiogenesis of vascular endothelial cells, thereby inhibiting cancer cell proliferation [90]. These authors suggested that *LINC00894* is stabilized by a METTL3-YTHDC2-dependent pathway and inhibits malignancy via the Hippo signaling pathway. Despite mixed evidence of METTL3’s relationship to PTC prognosis, its decreased levels in PTC, strong relationship to lncRNAs, and knowledge of its downstream pathways make it a potential target for future PTC therapies.

#### 3.2.2. METTL16

METTL16 is a methyltransferase demonstrated to be upregulated in malignancies such as hepatocellular carcinoma [92], breast cancer [93], and gastric cancer [94], promoting tumor growth, invasion, and metastasis. Generally, studies demonstrate a decreased expression of METTL16 in PTC [68,95]. In a 2024 study, Li et al. demonstrated that downregulation of METTL16 in PTC enhanced lipid metabolism and malignant progression, and that overexpression prevents PTC progression by cooperating with YTHDC2 to induce RNA decay in Stearoyl-CoA desaturase 1 (SCD1) cells [95]. This study suggests that a potential target for PTC therapy may be METTL16 or downstream targets. While X Wang et al. did find that expression levels of METTL16 were reduced in PTC, they did not find that METTL16 was related to PTC prognosis [68]. Conversely, to these two studies, Liu et al. found increased expression of METTL16 in PTC, with elevated expression levels associated with poorer prognosis as indicated by Kaplan–Meier survival curves [96]. While METTL16 expression levels may serve as a biomarker for PTC diagnosis and prognosis, the studies on this protein are limited, with confounding findings regarding its associated expression levels and PTC. Therefore, further studies are needed to clarify this relationship as well as its association with PTC prognosis.

#### 3.2.3. ZC3H13

ZC3H13 is a methyltransferase that has been involved in m6A RNA modification, affecting RNA stability, processing, and translation. The expression and function of ZC3H13 are altered in several cancers, including breast cancer [97], cervical cancer [98], and hepatocellular carcinoma [99], among others. The association between the expression level of ZC3H13 and tumor growth varies between malignancies, with ZC3H13 acting as a tumor suppressor in some cancers and thus its downregulation is associated with tumor growth, while the opposite is true in some other cancers. In PTC, ZC3H13 is downregulated, indicating that ZC3H13 generally has a tumor-suppressive role [67,69,70,100]. A 2013 study by Xie et al. showed that overexpression of ZC3H13 led to inhibition of cell proliferation, invasion, and migration whereas decreased expression of ZC3H13 led to increased proliferation, invasion, and migration [100]. The authors propose this occurs by means of ZC3H13-mediated degradation of *IQGAP1* mRNA. Furthermore, K Wang et al. found that ZC3H13 was related to the prognosis of differentiated TC and included it in their risk signature [69]. However, Hou et al. did not find that ZC3H13 was significantly related to PTC prognosis. Lastly, ZC3H13 was shown to be related to 10 out of 19 lncRNAs studied by W Wang et al.; however, prognostic associations of these associations were not noted [70]. As studies have generally found decreased expression of ZC3H13 to be associated with tumor size, metastasis, and prognosis of PTC, future studies should explore its potential as a therapeutic target and biomarker for PTC.

### 3.3. M6A Modifying Erasers

#### 3.3.1. FTO

Fat mass and obesity-associated protein (FTO) is an enzyme that removes RNA m6A modifications through its role as a demethylase [101]. Previous studies have demonstrated the role of FTO in cancer cell proliferation, metastasis, apoptosis, regulation of cancer stem cells, regulation of the tumor microenvironment, and drug resistance [102,103]. In malignancies such as gastric cancer [104] and breast cancer [105], upregulation of FTO has been associated with a worse prognosis. Conversely, FTO has been shown to act as a tumor suppressor, and its expression is decreased in PTC [67,68,70,74,89]. One study found that FTO works by inhibiting growth and glycolysis, likely by reducing the expression of APOE which acts to promote tumor growth via the IL-6/JAK2/STAT3 pathway [74]. Interestingly, this study found that *APOE* mRNA is stabilized by IGF2BP2 and that a knockdown of FTO significantly increased the binding of IGF2BP2 to *APOE* mRNA [74]. Another study has suggested that FTO works as a tumor suppressor by downregulating SLC7A11 in order to suppress metastasis [100]. In terms of the relationship of FTO to PTC prognosis, Hou et al. found that low levels of FTO were significantly related to poor prognosis of PTC and included it in its risk score [67]. Another study included FTO in its analysis of PTC survival and found that FTO was significantly related to the overall survival rate but was not related to disease-free survival in PTC [68]. In spite of this, this study did find that IGF2BP2 was related to disease-free survival, which has been shown to be involved in the downstream pathway of FTO [74]. FTO was also shown to be related to 11 out of 19 lncRNAs studied by W Wang et al., with no prognostic implications known [70]. While FTO has been associated with overall survival in PTC, its relationship with disease-free survival remains unclear, and the functional significance of its interaction with various lncRNAs is still unknown. Further research is needed to elucidate the full extent of FTO’s role in PTC progression and prognosis.

#### 3.3.2. ALKBH5

By removing m6A modifications on essential RNAs, ALKBH5 has been shown to be involved in the development and progression of several malignancies, such as breast [106], lung [22], colon [107], pancreatic [108], and more [109], with the prognostic association with expression level varying by cancer type. In PTC, several studies have found reduced expression levels of ALKBH5 as compared with controls [67,68,69,70]. One study found that performing a knockdown of ALKBH5 resulted in increased expression of circRNA nuclear receptor-interacting protein 1 (circNRIP1), promoting PTC cell glycolysis, proliferation, and tumorigenesis [110]. In terms of prognostic value, a study by K Wang et al. found ALKBH5 to be related to the prognosis of differentiated thyroid cancer and included it in its risk signature [69]. However, other studies did not find that ALKBH5 was related to PTC prognosis [67,68]. Several studies have established the relationship between the expression levels of ALKBH5 and the development of PTC, consistently demonstrating an inverse correlation between expression levels and prognosis. Future studies are warranted to determine if this relationship has any influence on drug uptake and overall survival.

#### 3.3.3. RBM15, KIAA1429, WTAP, YTHDF3

Other m6A regulators that have been found by some authors to be related to the prognosis of PTC include RBM15, KIAA1429, WTAP, and YTHDF3 [67,68,69]. However, the relationship between these m6A methylation regulators and prognosis in PTC is not consistent in the research and little further investigations into their significance in PTC have been performed.

## 4. Identification of M6A-Associated LncRNAs Involved in Papillary Thyroid Cancer and Their Prognostic Values

Research into m6A-related lncRNA involvement in PTC is sparse. One recent study utilized The Cancer Genome Atlas (TCGA) to identify 56 m6A-associated lncRNAs that are differently expressed in papillary thyroid cancer tissue in comparison to normal tissue [34]. In total, 3 out of these 56 m6A-associated lncRNAs were found to be significantly associated with disease-free survival: *RP11-423H2.3*, *RP11-20J15.3*, and *RP11-221N13.3*. The lncRNA *RP11-423H2.3* and *RP11-221N13.3* were both upregulated in high-risk groups, whereas *RP11-20J15.3* was downregulated in high-risk groups [34]. Using their unique prognostic signature, the authors found that these 3 lncRNAs could be used to accurately predict disease-free survival. The authors also investigated genes that had a significant relationship with these lncRNAs by using a co-expression network and found that *SYT12*, *SDC4*, and *CAMK2N1* had the most significant relationship with these 3 lncRNAs [34].

Another study by Huang et al. used the TCGA to identify 21 m6A-associated lncRNAs that are related to the prognosis of PTC [111]. They found that 20 of these lncRNAs were risk factors for poor prognosis while 1 was a protective factor. The authors used these lncRNAs to perform a cluster analysis and found that RAS, BRAF, and AJCC M stage were all significantly correlated to this analysis. Furthermore, they found relationships between these lncRNAs, the tumor immune microenvironment, and PD-L1. A prognostic model was then created using 3 of these m6A-related lncRNAs: *PSMG3-AS1*, *BHLHE40-AS1*, and *AC016747.3.* The expression levels of *PSMG3-AS1* and *BHLHE40-AS1* were found to correspond to 11 chemotherapy drugs. Increased expression of *PSMG3-AS1* led to increased sensitivity to mitomycin C, mitoxantrone, tegafur, idarubicin, gemcitabine, fulvestrant, and teniposide, and increased resistance to bosutinib. Increased expression of *BHLHE40-AS1* led to increased sensitivity to epirubicin, mitoxantrone, and doxorubicin and increased resistance to allopurinol.

W Wang et al. studied 20 m6A regulatory genes from TCGA database that are differently expressed in PTC, finding that most were positively correlated with lncRNA levels [70]. They created consensus clusters and found that these clusters were closely related to the TNM stage, histological subtype, T stage, extrathyroidal extension, and lymph node metastasis. A risk score was created using 8 m6A-related lncRNAs, which were found to be significantly related to overall survival and immune cell infiltration. The 8 m6A-related lncRNAs were as follows: *AC139795.2*, *TRAM2.AS1*, *POLR2J4*, *AC018653.3*, *DOCK9.DT*, *GABPB1.AS1*, *NORAD,* and *AL022328.2*. The authors found that their survival prediction model had a better ability to predict prognosis than TNM staging and noted that *NORAD* and *GABPB1-AS1* were the most meaningful lncRNA for further research due to their expression levels.

A study by Su et al. identified 70 m6A-related lncRNA that were prognostic for PTC. They formed 3 clusters [112]. The cluster with the worst prognosis had increased expression of CD8+ and CD4+ T cells whereas the cluster with best prognosis had increased expression of PD-L1 and CTLA-4. 11 m6A-related lncRNAs were used to create a risk model: *AC007365.1*, *AC008555.1*, *AC040160.1*, *AC064807.1*, *AC126773.4*, *AL023583.1*, *AL512306.2*, *EIF2AK3-DT*, *LINC00667*, *LYPLAL1-DT* and *MIR181A2HG*. Of these, it was found that decreased expression of *MIR181A2HG* inhibited proliferation and migration whereas increased *LYPLAL1-DT* expression led to proliferation and migration in PTC cells.

Overall, research into m6A-associated lncRNAs and their relationship to PTC is sparse; however, some studies do have encouraging data related to m6A-associated lncRNA-based survival predictions and drug sensitivities. Despite this, of the studies that have been conducted, there is little overlap in findings. More studies need to be conducted to determine which m6A-associated lncRNAs are in fact relevant for PTC and have a relationship to its prognosis.

## 5. Discussion

The identification of epigenetic modifications and their impact on malignancy development and progression provides an additional avenue for the identification and characterization of tumors. As early identification may be key to the complete treatment of cancers and prevention of aggressive behavior and/or recurrence, creating a test with the ability to detect several types of cancer may serve to fill the gap. Though several assays and modalities exist to assess epigenetic modifications ref. [113], the development of a methylation assay that could identify methylation abnormalities and assist in early detection of cancer could hence provide a broad screening method. As the role that epigenetics plays in several cancers, including TCs and more specifically PTC, are clarified, further identifying specific modifiers, as well as their modification targets could provide further clues and help clinicians in their treatment decisions.

Despite being small, the current body of research into the involvement of m6A-associated lncRNA in PTC suggests that m6A-associated lncRNA plays an important role in its pathogenesis and prognostic evaluation. Many m6A “readers”, “writers”, and “erasers” have been shown to be both tumorigenic and tumor suppressive in PTC like they are in a multitude of other cancers. Furthermore, the production of prognostic models centered on the expression of m6A-associated lncRNA that has occurred for many cancers has started to focus on PTC. Several studies identified by this review have created these prognostic or survival prediction models based on the presence of clusters of m6A-associated lncRNA and their significant relation to important tumor characteristics and prognostic factors. These models have the potential to considerably enhance the diagnosis and management of PTC as an adjunct to the established prognostic tools, TNM staging system, and TDS.

Although the current state of research on the prognostic value of m6A-associated lncRNA is promising, future research should be performed to further characterize and strengthen the prognostic models. This investigation may also allow for the development of treatments that target m6A-modified lncRNAs or their “writers”, “readers”, or “erasers” that have critical involvement in PTC pathogenesis. Novel therapeutics targeting m6A-associated lncRNAs may prove very useful because treatment options for PTC refractory to standard treatment and metastatic PTC are limited. First-line options for refractory or advanced PTC include local excision, radioactive iodine (RAI), and local ablation with modalities such as radiofrequency ablation [114,115]. Those patients who are unable to tolerate these modalities and are eligible for systemic therapy may receive chemotherapy, namely doxorubicin, or tyrosine-kinase inhibitors (TKIs). However, doxorubicin treatment has resulted in poor outcomes and carries a considerable side-effect profile, while TKIs have shown more promising results but adverse effects that some patients may not tolerate [114]. Patients requiring systemic therapies who are unable to receive or tolerate established systemic therapies warrant investigation into the production of other targeted therapies, such as those targeting m6A-lncRNA in PTC. Of these targeted therapies, the various “reader”, “writer”, and “eraser” enzymes are attractive enzymes to study, given their many established roles in other malignancies. While studies on these enzymes’ roles in PTC have been relatively limited, several proteins appear to influence the prognostic outcome of PTC. For instance, the associated finding of elevated levels of IGF2BP2 and differentiation and metastatic state make this reader a potential target for driving PTC into a better-differentiated state, thus promoting an indolent behavior and, in theory, a response to RAI. Similarly, the three writers, METTL3, METTL16, and ZC3H13 have all been shown to influence PTC progression, tumor size, and prognosis, and as the up- or down-regulation of the enzymes is associated with PTC, they may also be useful as biomarkers of disease. Lastly, the eraser ALKBH5 has demonstrated a consistent trend in expression levels and association with prognosis. As indicated in the discussion of findings, the studies on the role of these m6A-modifying enzymes in PTC have been limited. Of the studies that exist, most focus on the relationship between the enzyme’s expression levels in PTC, and any potential influence on tumor spread and prognosis. Little to no studies have examined the therapeutic potential of targeting these enzymes and their use as biomarkers to identify individuals at risk of more aggressive disease. While PTC is generally an indolent malignancy, its prevalence in younger patient populations, its tendency to recur, along with indications that ATC may stem from previously present PTC renders finding markers that can both prognosticate risk and serve as targets critical. The current biomarkers used to characterize PTC, such as BRAF, RAS, RET, and TERT, are insufficient prognosticators on their own, unable to identify these at-risk patients. M6A plays many well-established roles in malignancies; as we continue to understand how other parts of the genome, such as lncRNAs, influence factors resulting in tumorigenesis, more avenues for study and identification of specific targets become available. Ultimately, further studying the function of m6A, its modifying enzymes, and its influence on lncRNAs may help improve the diagnostic and prognostic approach to PTC, and its treatment.

## 6. Conclusions

M6A may play a driving role in the progression of PTC into a more severe phenotype which may increase the risk of patient cancer recurrence, and potential advancement to ATC. Further studies exploring the function of m6A, particularly as it relates to its modification of the lncRNAs known to interact in the pathogenesis of PTC, are needed, to further characterize its function. Thus, the presence of m6A modification of lncRNA in PTC may prove to be of clinical utility when assessing the phenotype of PTC, and may therefore factor into the clinical decision-making regarding treatment.

A limitation of this review is its narrative nature. While current literature on the role of m6A modification on PTC progression was examined, there was no systematic approach, which introduced the potential for bias in the literature. To mitigate this, the authors tried to provide a comprehensive overview and discussion that included all the major and relevant publications on the topic and addressed any contradictions in the literature where they existed.

## Figures and Tables

**Table 1:** Summary of the relationship between N6-Methyladenosine-modifying readers, writers, and erasers and papillary thyroid cancer.

	Upregulated or Downregulated	Role in PTC and Relationship to Prognosis	Number of Correlated lncRNA
HNRNPC	Upregulated	- No evidence for relationship to prognosis in PTC - Increased expression related to poor prognosis in DTC	3
IGF2BP2	Upregulated	- Increased expression related to poor prognosis and inversely related to disease free survival - Involved in differentiation and metastasis - Overexpression linked with decreased cisplatin sensitivity	9
YTHDC2	Downregulated	- Inhibits proliferation and apoptosis of PTC - No evidence for relationship to PTC prognosis	Unknown
METTL3	Downregulated	- Mixed results for relationship to PTC prognosis - Low levels correlated with poor immune checkpoint blockade - Overexpression linked with improved anti-PD-1 response	13, including *LINC00894*
METTL16	Mixed, mostly downregulated	- No evidence for relationship to PTC prognosis	Unknown
ZC3H13	Downregulated	- Inhibits cell proliferation, invasion, and migration - Mixed results for relationship to PTC prognosis	10
FTO	Downregulated	- Low levels related to poor prognosis and decreased overall survival rate - May be involved in same pathway as IGF2BP2	11
ALKBH5	Downregulated	- No evidence for relationship to prognosis in PTC - Decreased expression related to poor prognosis in DTC	12

## Data Availability

Data is contained within the article.

## Glossary

M6A	N6-Methyladenosine
TC	Thyroid Cancer
PTC	Papillary Thyroid Cancer
ATC	Anaplastic Thyroid Cancer
lncRNA	Long Non-coding RNA
TDS	Thyroid Differentiation Score
mRNA	MicroRNA
rRNA	Ribosomal RNA
miRNA	MicroRNA
circRNA	Circular RNA
snRNA	Small Nuclear RNA
HNRNPC	heterogeneous Nuclear Ribonucleoprotein C
IGF2BP2	Insulin-like Growth Factor 2 mRNA Binding Protein 2
YTHDC2	YTH N6-methyladenosine RNA Binding Protein C2
METTL3	Methyltransferase 3
METTL16	Methyltransferase 16
ZC3H13	Zinc Finger CCCH-Type Containing 13
FTO	Fat Mass and Obesity-Associated Protein
ALKBH5	AlkB Homolog 5
RBM15	RNA Binding Motif Protein 15
